# New 1-Aryl-3-Substituted Propanol Derivatives as Antimalarial Agents

**DOI:** 10.3390/molecules14104120

**Published:** 2009-10-14

**Authors:** Silvia Pérez-Silanes, Luis Berrade, Rory N. García–Sánchez, Adela Mendoza, Silvia Galiano, Berta Martín Pérez-Solórzano, Juan J. Nogal-Ruiz, Antonio R. Martínez-Fernández, Ignacio Aldana, Antonio Monge

**Affiliations:** 1 Unidad en Investigación y Desarrollo de Medicamentos, Centro de Investigación en Farmacobiología Aplicada (CIFA), University of Navarra, c/Irunlarrea s/n, 31080 Pamplona, Spain; E-Mail: luisberrade@msn.com (L.B.); 2 Departamento de Parasitología, Facultad de Farmacia, Complutense University of Madrid, Spain

**Keywords:** antimalarial agents, benzo[b]thiophene, 1-aryl-3-substituted propanol derivatives, *P. Falciparum*, ferriprotoporphyrin

## Abstract

This paper describes the synthesis and *in vitro* antimalarial activity against a *P. falciparum* 3D7 strain of some new 1-aryl-3-substituted propanol derivatives. Twelve of the tested compounds showed an IC_50_ lower than 1 µM. These compounds were also tested for cytotoxicity in murine J774 macrophages. The most active compounds were evaluated for *in vivo* activity against *P. berghei* in a 4-day suppressive test. Compound **12** inhibited more than 50% of parasite growth at a dose of 50 mg/kg/day. In addition, an FBIT test was performed to measure the ability to inhibit ferriprotoporphyrin biocrystallization. This data indicates that 1-aryl-3-substituted propanol derivatives hold promise as a new therapeutic option for the treatment of malaria.

## 1. Introduction 

Malaria is a major health disease with hundreds of millions of people being infected. Approximately 50% of the world’s population, mostly those living in the world’s poorest countries, are at risk of malaria [1]. Every year, more than 500 million people become severely ill with malaria. Most cases and deaths occur in tropical and subtropical countries. Early diagnosis and prompt treatment are the basic elements needed for the control of this condition. 

Chloroquine was the most effective and widely used drug in malaria therapy because of its rapid onset of action, good tolerability and low cost. However, the increasing resistance of the malaria parasite *Plasmodium falciparum* to currently available drugs, and especially to chloroquine, demands a continuous effort to develop new effective therapeutic options [2].

**Figure 1:**
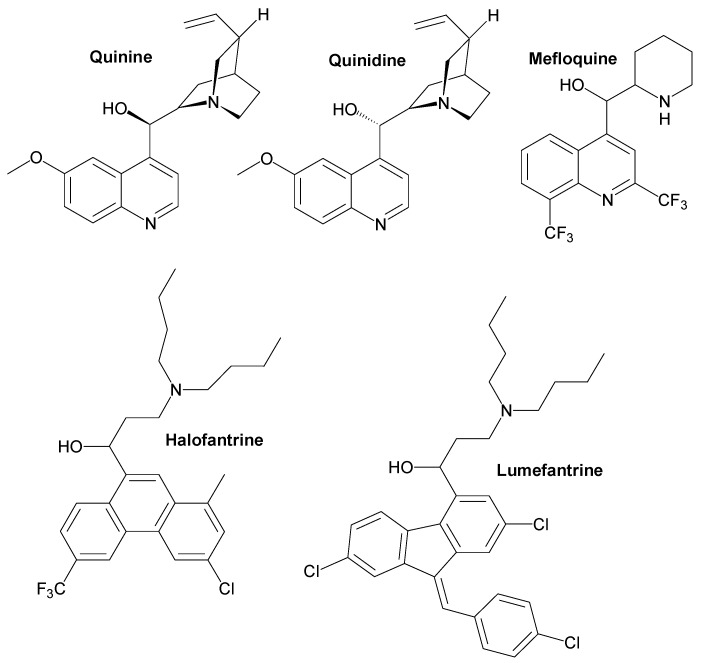
Antimalarial drugs: Arylaminealcohol derivatives.

The arylamine alcohols (Figure 1) are antimalarial drugs structurally derived from quinine. Mefloquine is considered a standard therapeutic agent for chloroquine-resistant malaria; however, its use is limited by high costs and the appearance of neuropsychiatric side effects [3]. The administration of halofantrine is highly effective, but has been severely restricted due to its potential to induce fatal adverse cardiac effects [4]. Lumefantrine, which is an analogue of halofantrine with no cardiac effects, is available coformulated with arthemether in an oral preparation [5].

Strains of *P. falciparum* that are resistant to chloroquine and many other antimalarial drugs have since emerged and this has created a near desperate situation, where the need for new, inexpensive antimalarial agents has become vital. Identification of new molecular scaffolds structurally unrelated to existing antimalarial agents represents a valuable strategy to bypass resistance phenomena.

More recently aminopropanol derivatives have been described as antimalarial agents [6,7,8], so we focused our attention on a series of 1-aryl-3-[4-arylpiperazin-1-yl]-1-propane derivatives synthesized by our group and recently published as antidepressants [9,10,11,12] (Figure 2). Our efforts have been focused on identifying new antimalarial drug candidates. As a result, we thought that the structural similarity of our compounds with the active arylamine alcohols in Figure 1 could also be interesting. Therefore, after several months of coordinated work between the synthesis and experimental chemotherapy, we report the synthesis and the antimalarial activity of 1-aryl-3-substituted propanol derivatives. We carried out both *in vitro* and *in vivo* assays in order to determine whether our compounds have potential for being considered as future antimalarial drugs. In addition, we performed an easy *in vitro* test in order to measure the ability of the compounds to inhibit the biomineralization process from ferriprotoporphyrin IX to hemozoin, as a possible mechanism of action.

## 2. Results and Discussion 

**Scheme 1:**
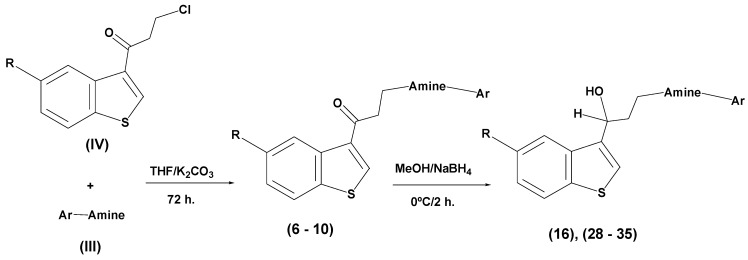
Synthesis of benzo[*b*]thiophenyl propanol derivatives **6-10**, **16** and **28-35**.

**Scheme 2:**
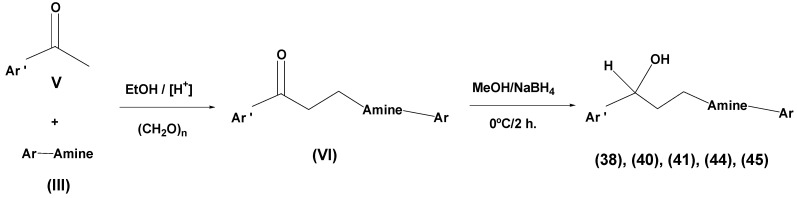
Synthesis of 1-aryl-3-substituted propanol derivatives **38, 40, 41, 44** and **45**.

**Scheme 3:**
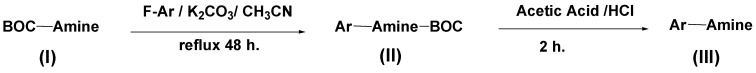
Synthesis of the non-commercially available arylamines.

The synthesis of some of the tested compounds has been published previously [9,10,11,12] (see reference publications in Table 1, Table 2 and Table 3. Methods for the synthesis of compounds **6-10**, **16**, **28-35**, **38**, **40**, **41**, **44** and **45** are presented in Scheme 1, Scheme 2 and Scheme 3, while derivatives **1-5**, **11-15**, **17-27**, **36**, **37**, **39**, **42** and **43** were previously reported [9,10,11].

The benzo[*b*]thiophene derivatives **6-10** were prepared using the 1-(3-benzo[*b*]thio phenyl)-3-chloropropan-1-one precursor **IV** previously synthesized by a Friedel-Craft acylation of benzo[*b*]thiophene as described in [12]. Ketones were obtained by a S_N_2 nucleophilic attack of the corresponding arylamine **III** to **IV** (Scheme 1).

The ketone intermediates **VI** were prepared by condensation of the corresponding commercially available acetophenones **V** with the different aryl amines **III** via Mannich reactions[12] (Scheme 2).

The 4-chloro-, 2-methoxy- and 3-methoxyphenyl piperazines were commercially available. The rest of the aryl amines were synthesized using the corresponding BOC-arylamines and *p*-nitrophenyl, *p*-nitro-*o*-trifluoromethylphenyl and *2*-quinoxalinyl aryl fluorides by an SNAr reaction [13] and subsequent removal of the BOC-group with HCl and acetic acid (Scheme 3). 

Finally, all the hydroxyl derivatives **11-46** were obtained by reduction of the corresponding carbonyl group with NaBH_4_ in methanol. All of the compounds were chemically characterized by thin layer chromatography (TLC), melting point (m.p.), infrared (IR) and nuclear magnetic resonance (^1^H-NMR) spectra, as well as by elemental microanalysis.

Forty-six compounds were evaluated for *in vitro* antimalarial activity against *P. falciparum*, and twelve of them showed good activity, with IC_50_ values lower than 1 µM. We paid special attention to compounds **12**, **15**, **22**, **23** and **31** since they were the most active, only two to fourfold less active than chloroquine. On the other hand, twenty-nine compounds showed moderate or low activity with IC_50_ values between 1 and 14.63 µM. All other compounds were considered to be inactive because they either showed no activity or their activity was quite insignificant (see Table 1 and Table 2). Chloroquine, the positive control, showed an IC_50_ of 0.08 µM, which coincides with the value reported by other authors.

From the point of view of the SAR and in order to assess the importance of the OH group present in the arylamine moiety, the *in vitro* antimalarial activity of some ketone intermediates (compounds **1-10**) against *P. falciparum* was evaluated. It is important to point out that none of them were active. This fact suggests that the OH group present in the arylamine moiety is essential for the activity. In addition, an increase in the activity was observed in the benzothiophene derivatives, when substituted in position 5 with an electron withdrawing group (R=F; compounds **12**, **15, 18**, **21**, **23**, **27**) in comparison with the corresponding unsubstituted derivatives (R=H; compounds **11**, **13**, **17**, **20**, **22**, **26**). Regarding the different amines employed, the activity data suggest that the most interesting compounds are those containing the 4-aminopiperidine moiety (see compounds **28-31** and **32-34**). Finally, it is evident that the presence of a benzocondensed group in Ar’ (benzothiophenyl or naftyl) contributes to a notable improvement in the activity compared to other aromatic substituents.

Before testing the *in vivo* antimalarial activity, we evaluated the cytoxicity of every active compound on a murine macrophages J774 cell line. Although the majority of them were toxic at 100 µg/mL, only compound **31** showed toxicity at 10 µg/mL. From our point of view, if a compound shows a toxicity percentage higher than 30% at this concentration, it will be toxic *in vivo*. However, we decided to include it in an *in vivo* test with mice (Table 3). Only compound **12** inhibited more than 50% of parasite growth and the appearance of the mice was good at the end of the experiment. The mice in groups treated with compounds **23** and **15** looked unhealthy and showed clear evidence of weight loss. Unfortunately, the cytotoxicity of compound **31** was confirmed in the *in vivo* test.

Ferriporotoporphyrin IX (FPIX) biocrystallization is a *Plasmodium*-specific process in which the toxic FPIX derived from the digestion of ingested haemoglobin is converted into an insoluble nontoxic crystalline species called haemozoin. This is the mechanism through which chloroquine exerts its antimalarial effect. Due to the structural similarity of our compounds with other antimalarial arylamine alcohol derivatives, we felt that it could be important to examine this. The results showed that no compound was active in the FBIT test. This fact supports the hypothesis that 1-aryl-3-substituted propanol derivatives, such as quinine, have a weaker binding coefficient to FP [14]. This was demonstrated by Deharo [15], who reported an IC_50_ = 324µM for quinine in the FBIT test. This fact is of great importance because the parasite has developed resistance to the majority of the antimalarials of the 4-aminoquinoline group, all of which inhibit the formation of haemozoin.

## 3. Experimental 

### 3.1. Biological evaluation

#### 3.1.1. *In vitro* antimalarial activity screening against Plasmodium falciparum 

The SYBR^©^GreenI-based micromethod [16] was followed for testing the antimalarial activity of the compounds. Erythrocytic stages of *P. falciparum* 3D7 strain, chloroquine sensitive, were maintained in RPMI 1640 culture medium supplemented with 0.5% Albumax II at 37 °C in an atmosphere with 5% CO_2_. An erythrocyte suspension, with initial 1% parasitemia and 4% hematocrit, was prepared using the aforementioned culture and then distributed into a 96-well plate, 50 µL per well. Next, stock solutions of each compound were prepared in DMSO and diluted in RPMI medium in order to obtain concentrations from 10 to 0.01 µg/mL. The final DMSO concentration was never higher than 0.1%. 50 µL of each prepared concentration were added per well. DMSO and chloroquine were included as a negative and positive control, respectively. All compounds and controls were placed in triplicate. The plate was incubated under the same conditions. After 48 h, the plate was removed from the incubator and frozen for at least 1 h at -70 °C and then thawed. Finally, 100 µL of SYBR^©^GreenI in lysis buffer (0.2 µL/mL) was added per well and shaken for 5 minutes or until no precipitated erythrocytes were observed. The plate was left to stand in the dark for 1 h at room temperature. Fluorescence intensity (FI) for each well was measured. The background of the nonparasitized erythrocytes was subtracted from each well tested. Percentage inhibition of the parasite growth for each concentration was calculated by using the following formula:

% inhibition = 100 × [(F.I.control – F.I.comp)/(F.I.control)]

IC_50_ values were estimated by plotting drug concentration versus percentage inhibition.

#### 3.1.2. Nonspecific cytotoxicity tests [17]

Murine J774 macrophages were maintained in RPMI 1640 medium supplemented with 10% FBS at 37 °C in a 5% CO_2_ atmosphere. First, in a flat bottom 96-well microplate, 100 µL of macrophage suspension in RPMI medium, containing 5 × 10^4^ cells, were distributed per well. The cells were allowed to attach for 24 h at 37 °C. Next, the medium was replaced by different concentrations of the compounds in 200 µL of medium, or DMSO at the same concentration as growth control, and the cells were exposed to the compounds solutions for another 24 h. Each concentration was assayed three times. Afterwards, the medium was eliminated and 100 µL/well of 3-(4,5-dimethythiazol-2-yl)-2,5-diphenyltetrazolium bromide (MTT) solution, 0.4 mg/mL in PBS, was added and the plates were returned to incubator for 1 h. The suspension was removed and the toxic effect of the compounds was assessed by the reduction of MTT to formazan crystals (as a cell viability indicator); said crystals were solubilized by adding 100 µL of DMSO. Finally, the optical density (OD) was measured at 595 nm and the toxicity percentage was calculated as follows:

% toxicity = [(O.D.control - O.D.comp)/(O.D.control)] × 100

#### 3.1.3. *In vivo* antimalarial activity

The *in vivo* antimalarial activity of the compounds was measured by the classical 4-day suppressive test [18]. Briefly, on day 0, groups of five NMRI male mice, weighing 20 ± 2 g each, were inoculated with 2 × 10^7^ red blood cells RBCs infected by erythrocytic stages of rodent malaria parasite *P. berghei* ANKA strain. Two hours later, each group of mice was treated intraperitoneally with a dose of 50 mg/kg of the selected compound, previously prepared in DMSO. Treatment was continued from day 1 to day 3, always at similar times. On day 4, Giemsa-stained thin blood smears from the tail of the mice were prepared and microscopically examined with 1,000× magnification. The mean of the parasitemia of each group was calculated in a total of 1,000 RBCs, and the growth inhibition percentage of parasite was estimated in relation to the control group, which received only the solvent of the products.

% inhibition = [(Par control – Par comp)/(Par control)] × 100

#### 3.1.4. Ferriprotoporphyrin IX biomineralization inhibition test (FBIT)

The procedure for testing FP biomineralization was carried out according to the method described by Deharo [15]. A mixture containing 50 µL of a 10 mg/mL drug solution or 50 µL of solvent (for control), 50 µL of 0.5 mg/mL of haemin chloride (Sigma H 5533) freshly dissolved in dimethylsulphoxide (DMSO) and 100 µL of 0.5 M sodium acetate buffer pH 4.4 was incubated in a non-sterile flat bottom 96-well plate at 37 °C for 18-24 hrs. After incubation, the plate was centrifuged at 1,600 g for 5 min and the supernatant was discarded. The remaining pellet was resuspended with 200 µL of DMSO in order to remove unreacted FP. The plate was then centrifuged once again and the supernatant discarded. The pellet (precipitate of ß-haematin), was dissolved in 150 µL of 0.1 M NaOH and the absorbance quantified at 405 nm with a microplate reader. The data was expressed as the percentage of inhibition of FP biomineralization, calculated using the following equation:

% inhibition = 100 × [(O.D.control - O.D.drug)/(O.D.control)]

### 3.2. Chemistry

The ^1^H-NMR spectra were recorded on a Bruker 400 Ultrashield (400 MHz) (Bruker, Rheinstetten, Germany), using TMS as internal standard and chloroform (CDCl_3_) or dimethyl sulfoxide- *d_6_* (DMSO-*d_6_)* as solvents; the chemical shifts are reported in ppm (δ), and coupling constant (*J*) values are given in Hertz (Hz). Signal multiplicities are represented by: s (singlet), bs (broad singlet), d (doublet), t (triplet), q (quadruplet), dd (double doublet) and m (multiplet). The IR spectra were registered on a Thermo Nicolet FT-IR Nexus Euro (Thermo, Madison, WI, USA) using KBr pellets; the frequencies are expressed in cm^-1^. Elemental microanalyses were obtained on an Elemental Analyzer (LECO CHN-900, Leco, Tres Cantos Spain) from vacuum-dried samples. The analytical results for C, H, and N were within ± 0.4 of the theoretical values. Alugram SIL G/UV254 (Layer: 0.2 mm) (Macherey-Nagel GmbH & Co. KG. Germany) was used for thin layer chromatography and silica gel 60 (0.040–0.063 mm) for column flash chromatography (Merck). Chemicals were purchased from E. Merck (Darmstadt, Germany), Scharlau (F.E.R.O.S.A., Barcelona, Spain), Panreac Química S.A. (Montcada i Reixac, Barcelona, Spain), Sigma-Aldrich Química, S.A., (Alcobendas, Madrid, Spain, ), Acros Organics (Janssen Pharmaceuticalaan, Geel, Belgium) and Lancaster (Bischheim-Strasbourg, France).

#### 3.2.1. General method for the synthesis of protected aryl amines **II**

A mixture of the aryl fluoride (1 eq), the protected amine **I** (1.2 eq), K_2_CO_3_ (1.2 eq) and CH_3_CN (50 mL) was heated at reflux for 48 hours. The solvent was removed under reduced pressure, the residue was dissolved in CH_2_Cl_2_ (50 mL) and washed with water (3 × 30 mL). The organic phase was dried with anhydrous Na_2_SO_4_ and filtered. After evaporating to dryness under reduced pressure, the residue was precipitated and washed by adding diethyl ether or petroleum ether, affording the desired protected aryl amine **II**.

#### 3.2.2. General method for the preparation of deprotected amines **III**

The protected amine **II** was dissolved in 40 mL of a solution of HCl/AcOH (1:1) with stirring for 2 hours at room temperature. The solvent was removed under reduced pressure and the compound was dissolved in water. The aqueous solution was basified with NaOH 2M to basic pH and stirred for 1 hour. Then the product was extracted with CH_2_Cl_2_. The organic phase was dried with anhydrous Na_2_SO_4_ and filtered. After evaporating to dryness under reduced pressure the residue was precipitated and washed by adding diethyl ether or petroleum ether, affording the desired deprotected amine **III**.

#### 3.2.3. General method for the synthesis of ketones **6-10**

A mixture of 1-(benzo[*b*]thiophen-3-yl)-3-chloropropan-1-one **IV** (1 eq), the arylamine **III** (1.2 eq) and K_2_CO_3_ (1.2 eq) was stirred in THF (50 mL) for 72 hours at room temperature. The solvent was removed under reduced pressure and the residue was dissolved in CH_2_Cl_2_ (40 mL) and washed with water (3 × 30 mL). After evaporating to dryness under reduced pressure the residue was purified by column chromatography (SP: silica gel), eluting with CH_2_Cl_2_/methanol 99:1 (v/v). In some cases the compound has been purified by preparative chromatography (SP: silica gel), eluting with CH_2_Cl_2_/methanol 95:5 (v/v). In the cases in which the hydrochloride salt was prepared, the process consisted in adding an ethereal hydrogen chloride solution to a dichloromethane solution of the compound.

#### 3.2.4. General method for the synthesis of ketones **VI**

A mixture of the appropriated substituted acetophenone **V** (1 eq), arylamine **III** or a commercially available one (1 eq) in absolute EtOH (40 mL) and concentrated HCl (pH = 2-3) was heated at reflux. Paraformaldehyde (3 eq) was added in four equal portions over a period of 40 min. The reaction mixture was refluxed for another 24 h, cooled and poured onto crushed ice. The separated solid was filtered, dried and recrystallized from 1-propanol.

#### 3.2.5. General method for preparation of hydroxyl derivatives **16**, **28-35**, **38**, **40**, **41**, **44** and **45**

Sodium borohydride (3 eq) was added little by little to a precooled suspension (0 °C, 5 min) of the corresponding ketone (1 eq) in methanol (40 mL) over a period of 30-60 minutes. The solvent was removed under reduced pressure and the residue dissolved in dichloromethane (40 mL) was washed with water (3 x 30 mL). The organic phase was dried with anhydrous Na_2_SO_4_ and filtered. After evaporating the solvent to dryness under reduced pressure, the compound was purified by column chromatography (SP: silica gel), eluting with CH_2_Cl_2_/methanol 99:1 (v/v) or by preparative chromatography (SP: silica gel), eluting with CH_2_Cl_2_/methanol 95:5 (v/v).

*1-(Benzo[b]thiophen-3-yl)-3-[4-(4-nitro-2-trifluoromethylphenyl)piperazin-1-yl]propan-1-ona* (**6**). Yield 3%; Mp 140-141 °C; ^1^H-NMR (CDCl_3_): δ 2.76-2.77 (m, 4H, **H_2_ + H_6_** piperazine); 3.02 (c, 2H, CO-CH_2_-C**H_2_**); 3.19-3.21 (t, 4H, **H_3_ + H_5_** piperazine); 3.27-3.30 (c, 1H, CO-C**H_2_**-CH_2_); 7.29 (dd, 1H, **H_6’_** phenyl, *J*_6’,5’_ = 9.4 Hz, *J*_6’,3’_ = 2.5 Hz ); 7.46 (t, 1H, **H_6_** benzothiophenyl, *J*_6,5_ = 8.0 Hz); 7.52 (t, 1H, **H_5_** benzothiophenyl, *J*_5,4_ = 8.1 Hz); 7.90 (d, 1H, **H_4_** benzothiophenyl, *J*_4,5_ = 8.0 Hz); 8.33-8.36 (m, 2H, **H_5’_** phenyl + **H_7_** benzothiophenyl); 8.53 (s, 1H, **H_2_** benzothiophenyl); 8.78 (d, 1H, **H_3’_** phenyl) ppm; Anal. Calcd. for (C_22_H_20_N_3_F_3_O_3_S) C, 57.02; H, 4.32; N, 9.07. Found: C, 56.80; H, 4.53; N, 9.06.

*1-(Benzo[b]thiophen-3-yl)-3-[3-(4-nitro-2-trifluoromethylphenylamino)-(R)-pyrrolidin-1-yl] propan-1-one* (**7**). Yield 5%; Mp 98-100 °C; ^1^H-NMR (CDCl_3_): δ 1.76-1.83 (m, 1H, **H_4ec_** pyrrolidine); 2.40-2.48 (m, 1H, **H_4ax_** pyrrolidine); 2.60 (dd, 1H, **H_5ax_** pyrrolidine); 2.80 (dd, 1H, **H_2ec_** pyrrolidine); 2.93 (dd, 1H, **H_2ax_** pyrrolidine); 3.00-3.10 (m, 3H, **H_5ec_** pyrrolidine + CO-CH_2_-C**H_2_**); 3.27 (t, 2H, CO-C**H_2_**, *J_CH2-CH2_* = 6.8 Hz); 4.16 (s.a, 1H, **H_3_** pyrrolidine); 5.37 (d, 1H, N**H**); 6.68 (d, 1H, **H_6’_** phenyl, *J*_6’,5’_ = 9.3 Hz); 7.45 (dd, 1H, **H_5_** benzothiophenyl, *J*_5,6_ = 7.1 Hz, *J*_5,4_ = 8.0 Hz, *J*_57_ = 1.1 Hz); 7.51 (dd, 1H, **H_6_** benzothiophenyl, *J*_6,5_ = 7.2 Hz, *J*_6,7_ = 8.2 Hz, *J*_6,4_ = 1.1 Hz); 7.88 (dd, 1H, **H_4_** benzothiophenyl, *J*_4,5_ = 8.0 Hz, *J*_46_ = 1.0 Hz); 8.22 (dd, 1H, **H_5’_** phenyl, *J*_5’,6’_ = 9.3 Hz, *J*_5’,3’_ = 2.3 Hz); 8.36 (s, 1H, **H_2_** benzothiophenyl); 8.39 (d, 1H, **H_3_**_’_ phenyl, *J*_3’,5’_ = 2.5 Hz); 8.77 (dd, 1H, **H_7_** benzothiophenyl, *J*_7,6_ = 8.2 Hz, *J*_7,5_ = 1.0 Hz) ppm; Anal. Calcd. for (C_22_H_20_N_3_F_3_O_3_S) C, 57.02; H, 4.32; N, 9.07. Found: C, 56.72; H, 4.32; N, 9.22.

*1-(Benzo[b]thiophen-3-yl)-3-[1-(4-nitrophenyl)piperidin-4-ylamino]propan-1-one* (**8**). Yield 57%; Mp 144-145 °C; ^1^H-NMR (DMSO-d_6_): δ 1.22-1.31 (m, 2H, **H_3ax_** + **H_5ax_** piperidine); 1.79 (bs, 1H, N**H**); 1.78 (dd, 2H, **H_3ec_** + **H_5ec_** piperidine); 2.71-2.78 (m, 1H, **H_4_** piperidine); 2.95 (t, 2H, CO-C**H_2_**, *J_CH2-CH2_* = 6.6 Hz); 3.05-3.11 (m, 2H, **H_2ax_ + H_6ax_** piperidine); 3.18 (t, 2H, C**H_2_**-NH, *J_CH2-CH2_* = 6.6 Hz); 3.92 (d, 2H, **H_2ec_ + H_6ec_** piperidine); 7.00 (d, 2H, **H_2’_** + **H_6’_** phenyl, *J*_2’,3’_ = *J*_6’,5’_ = 9.6 Hz); 7.43-7.52 (m, 2H, **H_5_** + **H_6_** benzothiophenyl); 8.02 (d, 2H, **H_3’_** + **H_5’_** phenyl, *J*_3’,2’_ = *J*_5’,6’_ *=* 9.5 Hz); 8.08 (d, 1H, **H_4_** benzothiophenyl); 8.61 (d, 1H, **H_7_** benzothiophenyl); 8.97 (s, 1H, **H_2_**) ppm; Anal. Calcd. for (C_22_H_23_N_3_O_3_S) C, 64.53; H, 5.66; N, 10.26. Found: C, 64.88; H, 5.67; N, 10.28. 

*Hydrochloride of 1-(benzo[b]thiophen-3-yl)-3-[4-(4-nitrophenyl)-1,4-diazepan-1-yl]propan-1-one* (**9**). Yield 57%; Mp 121-123 °C; ^1^H-NMR (DMSO-d_6_): δ 2.22-2.28 (m, 1H, **H_6_** diazepane); 2.43-246 (m, 1H, **H_6_** diazepane); 3.20-3.25 (m, 2H, **H_7_** diazepane); 3.35 (m, 2H, CO-CH_2_-C**H_2_**); 3.53-3.56 (m, 2H, **H_2_** diazepane); 3.63-3.67 (m, 2H, **H_5_** diazepane); 3.70-3.72 (m, 2H, CO-C**H_2_**); 3.87-3.95 (m, 1H, **H_3_** diazepane); 4.03-4.09 (m, 1H, **H_3_** diazepane); 6.92(d, 2H, **H_2’_** + **H_6’_** phenyl, *J*_2’,3’_ = *J*_6’,5’_ = 9.4 Hz); 7.46-7.55 (m, 2H, **H_6_ + H_5_** benzothiophenyl); 8.08-8.12 (m, 3H, **H_4_** benzothiophenyl**+ H_3_’+ H_5_’** phenyl); 8.60 (d, 1H, **H_7_** benzothiophenyl, *J*_7,6_ = 8.1 Hz); 9.06 (s,1H, **H_2_** benzothiophenyl); 10.95 (s.a., 1H, **H**Cl) ppm; Anal. Calcd. for (C_22_H_23_N_3_O_3_S·HCl) C, 59.25; H, 5.42; N, 9.42. Found: C, 58.86; H, 5.69; N, 9.08.

*1-(Benzo[b]thiophen-3-yl)-3-[4-(quinoxalin-2-yl)piperazin-1-yl]propan-1-one* (**10**). Yield 13%; Mp 145-146 °C; ^1^H-NMR (CDCl_3_): δ 2.74 (t, 4H, **H_2_ + H_6_** piperazine); 3.00 (t, 2H, CO-CH_2_-C**H_2_**); 3.08 (t, 2H, CO-C**H_2_**); 3.85 (t, 4H, **H_3_ + H_5_** piperazine); 7.40-7.47 (m, 2H, **H_5_** benzothiophenyl **+ H_7’_** quinoxalinyl); 7.53 (dd, 1H, **H_6_** benzothiophenyl, *J*_6,5_ = 8.3 Hz, *J*_6,7_ = 7.1 Hz, *J*_6,4_ = 1.2 Hz); 7.60 (m, 1H, **H_6’_** quinoxalinyl); 7.71 (dd, 1H, **H_5’_** quinoxalinyl, *J*_5’,6’_ = 8.4 Hz, *J*_5’,7’_ = 1.3 Hz); 7.91 (m, 2H, **H_8’_** quinoxalinyl **+ H_4_** benzothiophenyl); 8.38 (s, 1H, **H_2_** benzothiophenyl); 8.60 (s, 1H, **H_3’_**quinoxalinyl); 8.79 (dd, 1H, **H_7_** benzothiophenyl, *J*_7,6_ = 8.2 Hz, *J*_7,5_ = 0.7 Hz ) ppm; Anal. Calcd. for (C_23_H_22_N_4_OS) C, 68.63; H, 5.51; N, 13.92. Found: C, 68.58; H, 5.42; N, 13.77. 

*Dihydrochloride of 1-(5-fluorobenzo[b]thiophen-3-yl)-3-[4-(3-methoxyphenyl)piperazin-1-yl]propan-1-ol* (**16**). Yield 42%; Mp 135-137 °C; ^1^H-NMR (DMSO-d_6_): δ 2.22-2.24 (m, 2H, CHOH-C**H**_2_); 3.12-3.16 (m, 4H, **2H_2_ + 2H_6_** piperazine); 3.28-3.33 (m, 2H, CHCH_2_C**H_2_**); 3.56-3.58 (m, 2H, **1H_3ec_**+**1H_5ec_** piperazine); 3.73 (s, 3H, OC**H_3_**); 3.79-3.82 (m, 2H, **H_3ax_+H_5ax_** piperazine); 4.52 (bs, 1H, O**H**); 5.03 (t, 1H, C**H**OH, *J_CH-CH2_* = 7.6 Hz); 6.44 (d, 1H, **H_4’_** phenyl, *J*_4’,5’_ = 8.0 Hz, *J*_4’,6’_ = 2.0 Hz); 6.46 (s, 1H, **H_2’_** phenyl); 6.52 (d, 1H, **H_6’_** phenyl, *J*_6’,5’_ = 8.0 Hz, *J*_6’,4’_ = 2.0 Hz); 7.13 (t, 1H, **H_5’_** phenyl, *J*_5’,6’_ = *J*_5’,4’_ = 8.4 Hz); 7.27 (t, 1H, **H_6_** benzothiophenyl, *J*_6,7_ = 8.8 Hz, *J*_6,4_ = 2.4 Hz); 7.77 (s, 1H, **H_2_** benzothiophenyl); 7.81 (d, 1H, **H_7_** benzothiophenyl, *J*_7,6_ = 8.8 Hz); 8.03 (dd, 1H, **H_4_** benzothiophenyl, *J* = 8.8 Hz)); 10.91 (s, 1H, OH); ppm; Anal. Calcd. for (C_22_H_25_FN_2_O_2_S. 2HCl) C, 55.81; H, 5.70; N, 5.91. Found: C, 55.41; H, 5.82; N, 5.43. 

*1-(Benzo[b]thiophen-3-yl)-3-[4-(4-nitro-2-trifluoromethylphenyl)piperazin-1-yl]propan-1-ol* (**28**). Yield 3%; Mp 154-155 °C; ^1^H-NMR (CDCl_3_): 2.14-2.16 (m, 2H, CHOH-C**H_2_**); 2.77-2.87 (m, 6H, CHOH-CH_2_**-**C**H_2_ + H_2_+H_6_** piperazine); 3.20-3.29 (m, 4H, **H_3_+ H_5_** piperazine); 5.38 (t, 1H, C**H**OH, *J_CH-CH2_* = 7.5 Hz); 7.33-7.42 (m, 3H, **H_6’_** phenyl *+* **H_6_** *+* **H_5_** benzothiophenyl); 7.45 (s, 1H, **H_2_** benzothiophenyl); 7.82 (d, 1H, **H_4_** benzothiophenyl, *J*_4,5_ = 8.4 Hz); 7.88 (d, 1H, **H_7_** benzothiophenyl, *J*_7,6_ = 8.0 Hz); 8.37 (dd, 1H, **H_5’_** phenyl *J*_5’,6’_ = 9.2 Hz, *J*_5’,3’_ = 2.8 Hz); 8.55 (d, 1H, **H_3’_** phenyl) ppm; Anal. Calcd. for (C_22_H_22_N_3_F_3_O_3_S) C, 57.77; H, 4.73; N, 9.03. Found: C, 57.01; H, 4.85; N, 8.54. 

*1-(Benzo[b]thiophen-3-yl)-3-[3-(4-nitro-2-trifluoromethylphenylamino)-(R)-pyrrolidin-1-yl]propan-1-ol* (**29**). Yield 47%; Mp 48-50 °C; ^1^H-NMR (DMSO-d_6_): δ 1.82-1.89 (m, 1H, **H_4ec_** pyrrolidine); 2.06-2.21 (m, 2H, CHOH-C**H_2_**); 2.47-2.56 (m, 1H, **H_4ax_** pyrrolidine); 2.76-2.85 (m, 3H, CHOH-CH_2_-C**H**_2_ + **H_2ec_ + H_5ax_** pyrrolidine); 3.00-3.15 (m, 2H, CHOH-CH_2_-C**H**_2_ **+ H_5ec_** pyrrolidine); 3.07-3.11 (m, 1H, **H_2ax_** pyrrolidine); 4.18-4.26 (m, 1H, **H_3_** pyrrolidine); 5.27 (bs, 1H, N**H**); 5.36-5.38 (m, 1H, C**H**OH); 6.72 (dd, 1H, **H_6’_** phenyl, *J*_6’,5’_ = 9.3 Hz, *J*_6’,3’_ = 2.3 Hz); 7.34-7.40 (m, 2H, **H_5_**, **H_6_** benzothiophenyl); 7.44 (d, 1H, **H_2_** benzothiophenyl, *J* = 7.3 Hz); 7.80-7.83 (m, 1H, **H_4_** benzothiophenyl); 7.87-7.90 (m, 1H, **H_7_** benzothiophenyl); 8.29 (dd, 1H, **H_5’_** phenyl, *J*_5’,6’_ = 9.4 Hz, *J*_5’,3’_ = 2.2 Hz); 8.44 (t, 1H, **H_3’_** phenyl, *J*_3’,F_ = *J*_3’,5’_ = 2.9 Hz) ppm; Anal. Calcd. for (C_22_H_22_N_3_F_3_O_3_S) C, 56.77; H, 4.76; N, 9.07. Found: C56.50; H, 4.88; N, 9.02. 

*1-(Benzo[b]thiophen-3-yl)-3-[1-(4-nitro-2-trifluoromethylphenyl)-(S)-pyrrolidin-3-ylamino]propan-1-ol* (**30**). Yield 18%; Mp 109-110 °C;. ^1^H-NMR (CDCl_3_): δ 1.94-2.07 (m, 2H, CHOH-C**H_2_**); 2.15-2.20 (m, 1H, **H_4ec_** pyrrolidine); 2.24-2.31 (m, 1H, **H_4ax_** pyrrolidine); 2.91-3.00 (m, 1H, CHOH-CH_2_-C**H**_2_); 3.04-3.10 (m, 1H, CHOH-CH_2_-C**H**_2_) 3.20 (bs, 1H, O**H**); 3.41 (td, 1H, **H_2ec_** pyrrolidine); 3.49-3.54 (m, 1H, **H_5ec_** pyrrolidine); 3.58-3.65 (m, 1H, **H_2ax_** pyrrolidine); 3.72-3.80 (m, 2H, **H_5ax_** + **H_3_** pyrrolidine); 5.35 (bs, 1H, C**H**OH); 6.84 (dd, 1H, **H_6’_,** *J*_6’,5’_ = 9.5 Hz, **H_6’_** phenyl, *J*_6’,3’_ = 2.5 Hz); 7.33-7.43 (m, 3H, **H_5_** + **H_6_** + **H_2_** benzothiophenyl); 7.77-7.80 (m, 1H, **H_4_** benzothiophenyl); 7.87-7.89 (m, 1H, **H_7_** benzothiophenyl); 8.18-8.22 (m, 1H, **H_5’_** phenyl); 8.58 (dd, 1H, **H_3’_** phenyl, *J*_3’,5’_ = 5.1 Hz, *J*_3’,6’_ = 2.6 Hz) ppm; Anal. Calcd. for (C_22_H_22_N_3_F_3_O_3_S) C, 56.77; H, 4.76; N, 9.07. Found: C, 56.33; H, 4.94; N, 9.06. 

*1-(Benzo[b]thiophen-3-yl)-3-[1-(4-nitro-2-trifluoromethylphenyl)piperidin-4-ylamino]propan-1-ol* (**31**). Yield 33%; Mp 145-146 °C; ^1^H-NMR (CDCl_3_): δ 1.57-1.68 (m, 2H, **H_3ax_** + **H_5ax_** piperidine); 1.94-2.03 (m, 1H, CHOH-C**H**_2_); 2.08 (d, 2H, **H_3ec_** + **H_5ec_** piperidine); 2.15-2.20 (m, 1H, CHOH-C**H**_2_); 2.71-2.77 (m, 1H, **H_4_** piperidine); 2.91-3.01 (m, 3H, **H_2ax_** + **H_6ax_** piperidine + C**H**_2_-NH); 3.07-3.12 (m, 1H, C**H**_2_-NH); 3.41 (d, 2H, **H_2ec_** + **H_6ec_** piperidine); 5.38 (m, 1H, C**H**OH); 7.27 (d, 1H, **H_6’_** phenyl, *J*_6’,5’_ = 8.9 Hz); 7.34-7.40 (m, 2H, **H_5_** + **H_6_** benzothiophenyl**)**; 7.46 (s, 1H, **H_2_** benzothiophenyl); 7.81 (d, 1H, **H_4_**, benzothiophenyl *J*_4,5_ *=* 7.9 Hz); 7.88 (d, 1H, **H_7_** benzothiophenyl, *J*_7,6_ *=* 7.8 Hz); 8.32 (dd, 1H, **H_5’_** phenyl, *J*_5’,6’_ = 9.0 Hz, *J*_5’,3’_ *=* 2.3 Hz); 8.53 (d, 1H, **H_3’_** phenyl, *J*_3’,5’_ = 2.4 Hz) ppm; Anal. Calcd. for (C_23_H_24_N_3_F_3_O_3_S) C, 57.61; H, 5.04; N, 8.76. Found: C, 57.39; H, 5.06; N, 8.80. 

*1-(5-Fluorobenzo[b]thiophen-3-yl)-3-[4-(4-nitrophenyl)piperazin-1-yl]propan-1-ol* (**32**). Yield 14%; Mp 189-190 °C; ^1^H-NMR (CDCl_3_): δ 1.60 (bs, 1H, O**H**); 2.10-2.15 (m, 2H, CHOH-C**H_2_**); 2.79-2.90 (m, 6H, CHOH-CH_2_**-**C**H_2_ + H_2_+H_6_** piperazine); 3.53 (t, 4H, **H_3_+ H_5_** piperazine); 5.30 (dd, 1H, C**H**OH); 6.88 (d, 2H, **H_2’_+ H_6’_** phenyl, *J*_2’,3’_ = *J*_6’,5’_ = 9.4 Hz); 7.14 (td, 1H, **H_6_** benzothiophenyl, *J*_6,7_ = *J*_6,F_ = 8.7 Hz, *J*_6,4_ = 2.4 Hz); 7.50-7.53 (m, 2H, **H_2_ + H_4_** benzothiophenyl); 7.81 (dd, 1H, **H_7_** benzothiophenyl, *J*_7,6_ = 8.8 Hz, *J*_7,F_ = 4.8 Hz); 8.17 (d, 2H, **H_3_’+ H_5_’** phenyl, *J*_3’,4’_ = *J*_5’,6’_ = 9.4 Hz) ppm; Anal. Calcd. for (C_21_H_22_N_3_FO_3_S) C, 60.71; H, 5.34; N, 10.11. Found: C, 60.44; H, 5.43; N, 9.79. 

*1-(Benzo[b]thiophen-3-yl)-3-(1-(4-nitrophenyl)piperidin-4-ylamino)propan-1-ol* (**33**). Yield 57%; Mp 153-155 °C; ^1^H-NMR (DMSO-d_6_): δ 1.23-1.31 (m, 2H, **H_3ax_** + **H_5ax_** piperidine); 1.86-1.96 (m, 4H, **H_3ec_** + **H_5ec_** piperidine + CHOH-C**H_2_**); 2.69-2.73 (m, 3H, **H_2ax_** + **H_6ax_** + **H_4_** piperidine); 3.07 (t, 2H, C**H_2_**-NH); 3.95 (d, 2H, **H_2ec_** + **H_6ec_** piperidine); 5.08 (s.a., 1H, C**H**OH); 7.27 (d, 1H, **H_2’_**+ **H_6’_** phenyl, *J*_2’,3’_ = *J*_6’,5’_ = 8.7 Hz); 7.33-7.39 (m, 2H, **H_5_** + **H_6_** benzothiophenyl); 7.53 (s, 1H, **H_2_** benzothiophenyl); 7.93-7.97 (m, 2H, **H_4_** + **H_7_** benzothiophenyl); 8.32 (dd, 1H, **H_3’_**+ **H_5’_** phenyl, *J*_3’,2’_ *= J*_5’,6’_ = 8.8 Hz) ppm; Anal. Calcd. for (C_22_H_25_N_3_O_3_S) C, 64.21; H, 6.12; N, 10.21. Found: C, 64.00; H, 5.97; N, 10.43. 

*1-(Benzo[b]thiophen-3-yl)-3-(4-(4-nitrophenyl)-1,4-diazepan-1-yl)propan-1-ol* **(34)**. Yield 61%; Mp 47-48 °C; ^1^H-NMR (DMSO-d_6_): δ 2.00-2.16 (m, 4H, **2H_6_** diazepane + CHOH-C**H_2_**); 2.68-3.00 (m, 6H, CH-CH_2_-C**H_2_** + **2H_2_ + 2H_7_** diazepane); 3.64-3.68 (m, 2H, **2H_5_** diazepane); 3.74 (bs, 2H, **2H_3_** diazepane); 5.32-5.38 (m, 1H, C**H**OH); 6.67 (d, 2H, **H_2’_+ H_6’_** phenyl, *J*_2’,3’_ = *J*_6’,5’_ = 9.4 Hz); 7.36-7.39 (m, 2H, **H_6_+H_5_** benzothiophenyl); 7.43 (s, 1H, **H_2_** benzothiophenyl); 7.78 (d, 1H, **H_4_**, *J_4,5_* = 7.42 benzothiophenyl); 7.88 (d, 1H, **H_7_** benzothiophenyl, *J_7,6_* = 7.03 Hz); 8.15 (d, 2H, **H_3’_+H_5’_** phenyl, *J*_3’,2’_ *= J*_5’,6’_ = 9.0 Hz) ppm; Anal. Calcd. for (C_22_H_25_N_3_O_3_S) C, 65.23; H, 6.08; N, 10.22. Found: C, 65.44; H, 6.45; N, 9.79.

*1-(Benzo[b]thiophen-3-yl)-3-(4-(quinoxalin-2-yl)piperazin-1-yl)propan-1-ol* (**35**). Yield 15%; Mp 155-156 °C; ^1^H-NMR (DMSO-*d*_6_): δ 2.29-2.32 (m, 2H, CHOHC**H_2_**); 3.13-3.22 (m, 6H, CH-CH_2_-C**H_2_ + 2H_2_ + 2H_6_** piperazine); 3.46-3.54 (m, 2H, **H_3_** piperazine); 3.56-3.64 (m, 2H, **H_5_** piperazine); 5.05-5.07 (m, 1H, C**H**OH); 5.76 (bs, 1H, O**H**); 7.37-7.44 (m, 2H, **H_5_ + H_6_** benzothiophenyl); 7.45-7.49 (m, 1H, **H_7’_** quinoxalinyl); 7.63-7.66 (m, 3H, **H_2_** + **H_4_** benzothiophenyl **+ H_6’_** quinoxalinyl); 8.88 (d, 1H, **H_7_** benzothiophenyl, *J*_7,6_ =8.4); 7.99 (dd, 2H, **H_5’_ + H_8’_** quinoxalinyl, *J*_5’,6’_ = *J*_8’,7’_ = 8.9 Hz, *J*_5’,7’_=*J*_8’,6’_ = 1.2 Hz); 8.90 (s, 1H, **H_3’_** quinoxalinyl) ppm; Anal. Calcd. for (C_23_H_24_N_4_OS) C, 68.31; H, 5.94; N, 13.86. Found: C, 68.70; H, 6.22; N, 13.65. 

*1-(4-Phenyl)phenyl -3-[4-(4-chlorophenyl)piperazin-1-yl]propan-1-ol* (**38**). Yield 47%; Mp 169-170 °C; ^1^H-NMR (DMSO-*d*_6_): δ 1.80-1.85 (m, 2H, CHOHC**H**_2_); 3.12-3.16 (m, 6H, CH-CH_2_-C**H_2_ +** 2**H_2_ +** 2**H_6_** piperazine); 3.34 (bs, 4H, 2**H_3_ +** 2**H_5_** piperazine); 4.68-4.70 (m, 1H, C**H**OH); 5.51 (s, 1H, O**H**); 6.93 (d, 2H, **H_2_+H_6_** Cl-phenyl, *J* = 9.0 Hz); 7.22 (d, 2H, **H_3_+H_5_** Cl-phenyl, *J* = 8.8 Hz); 7.35 (t, 1H, **H_4’_** phenyl, *J* = 8.4 Hz); 7.31-7.38 (m, 4H, **H_2_+H_6_** p-phenylphenyl + **H_3’_+H_5’_** phenyl); 7.61-7.67 (m, 4H, **H_3_+H_5_** p-phenylphenyl + **H_2’_+H_6’_** phenyl); ppm; Anal. Calcd. for (C_25_H_27_ClN_2_O) C, 73.80; H, 6.64; N, 6.88. Found: C, 73.48; H, 6.89; N, 6.78. 

*1-(4-Phenyl)phenyl-3-[4-(2-methoxyphenyl)piperazin-1-yl]propan-1-ol* (**40**). Yield 77% yield; Mp 109-110 °C; ^1^H-NMR (DMSO-*d*_6_): δ 1.79-1.83 (m, 2H, CHOHC**H**_2_); 2.38-2.54 (m, 6H, CHCH_2_C**H_2_ + 2H_2_ + 2H_6_** piperazine); 2.96 (bs, 4H, **2H_3_ + 2H_5_** piperazine); 3.76 (bs, 3H, OC**H_3_**); 4.70 (t, 1H, C**H**OH); 5.52 (bs, 1H, O**H**); 6.87-6.93 (m, 4H, **H_3_+H_4_+H_5_+H_6_** OC**H_3_**-phenyl); 7.35 (t, 1H, **H_4’_** phenyl); 7.42-7.48 (m, 4H, **H_2_+H_6_** p-phenylphenyl + **H_3’_+H_5’_** phenyl); 7.61-7.67 (m, 4H, **H_3_+H_5_** p-phenylphenyl + **H_2’_+H_6’_** phenyl); ppm; Anal. Calcd. for (C_26_H_30_N_2_O_2_) C, 77.61; H, 7.46; N, 6.96. Found: C, 77.58; H, 7.75; N, 6.84. 

*1-(3-Indolyl)-3-[4-(2-methoxyphenyl)piperazin-1-yl]propan-1-ol* (**41**). Yield 30% yield; Mp 188-189 °C; ^1^H-NMR (DMSO-*d*_6_): δ 1.91-2.03 (m, 2H, CHOHC**H**_2_); 2.38-2.51 (m, 6H, CHCH_2_C**H_2_ + 2H_2_ + 2H_6_** piperazine); 2.96 (bs, 4H, **2H_3_ + 2H_5_** piperazine); 3.76 (s, 3H, OC**H_3_**); 4.92 (t, 1H, C**H**OH); 5.16 (bs, 1H, O**H**); 6.87-6.98 (m, 5H, **H_3’_+H_4’_+H_5’_+H_6’_** phenyl + **H_6_** indolyl); 7.04-7.06 (t, 1H, **H_5_** indolyl, *J*_5,6_ = 8.0 Hz, *J*_5,4_ = 7.4 Hz); 7.20 (s, 1H, **H_2_** indolyl); 7.33 (d, 1H, **H_7_** indolyl, J_7,6_ = 8.4 Hz); 7.63 (d, 1H, **H_4_** indolyl, J_4,5_ = 7.4 Hz); 10.83 (s, 1H, N**H**) ppm; Anal. Calcd. for (C_22_H_27_N_3_O_2_) C, 72.33; H, 7.39; N, 11.51. Found: C, 72.23; H, 7.85; N, 11.19. 

*Dihydrochloride of 1-(6-methylnapth-2-yl)-3-[4-(2-methoxyphenyl) piperazin-1-yl]propan-1-ol* (**44**). Yield 35%; M.p.: 116-117 °C; ^1^H-NMR (DMSO-*d*_6_): δ 2.10-2.19 (m, 2H, CHC**H**_2_); 2.47 (s, 3H, CH_3_); 2.95-3.02 (m, 2H, **H_2ax_ + H_6ax_** piperazine); 3.16-3.29 (m, 4H, **H_2ec_ + H_6ec_** piperazine + CHCH_2_C**H_2_**); 3.45-3.57 (m, 4H, **H_3_ + H_5_** piperazine); 3.78 (s, 3H, OC**H_3_**); 4.80-4.83 (m, 1H, CH); 6.91-7.05 (m, 4H, **H_3’_+H_4’_+H_5’_+H_6’_** phenyl); 7.36 (d, 1H, **H_7_** naphthyl, *J*_7,8_ = 8.4 Hz); 7.50 (d, 1H, **H_3_** naphthyl, *J*_3,4_ = 8.8 Hz); 7.67 (s, 1H, **H_5_** naphthyl); 7.81 (m, 3H, **H_1_+H_4_+H_8_** naphthyl) ppm; Anal. Calcd. for (C_25_H_30_N_2_O_2_. 2HCl) C, 64.79; H, 6.91; N, 6.04. Found: C, 64.62; H, 7.05; N, 5.88. 

*Hydrochloride of 1-(2-naphthyl)-3-[3-(4-nitro-2-trifluoromethylphenyl)-(S)-pyrrolidin-3-yl amino]-propan-1-ol* (**45**). Yield 20%; Mp.: 181-183 °C; ^1^H-NMR (DMSO-*d*_6_): δ 2.07-2.14 (m, 2H, CHOH-C**H_2_**); 2.29-2.31 (m, 1H, **H_4ec_** pyrrolidine); 2.36-2.41 (m, 1H, **H_4ax_** pyrrolidine); 3.11 (bs, 2H, CHOH-CH_2_-C**H_2_**); 3.57-3.64 (m, 1H, **H_2ec_** pyrrolidine); 3.75-3.79 (m, 2H, **H_5ec_**+ **H_2ax_** pyrrolidine); 3.85-3.89 (m, 1H, **H_5ax_** pyrrolidine); 3.97 (bs, 1H, **H_3_** pyrrolidine); 5.90 (bs, 1H, C**H**OH); 5.76 (s, 1H, O**H**); 7.10 (d, 1H, **H_6’_** phenyl, *J*_6’,5’_ = 9.6); 7.47-7.55 (m, 3H, **H_3_** + **H_6_** + **H_7_** naphthyl); 7.86 (s, 1H, **H_1_** naphthyl); 7.90-7.92 (m, 3H, **H_4_** + **H_5_** + **H_8_** naphthyl); 8.24-8.9 (d, 1H, **H_5’_** phenyl, *J*_5’,6’_ = 9.5); 8.40 (s, 1H, **H_3’_** phenyl); 9.49 (bs, 1H, N**H**) ppm; Anal. Calcd. for (C_24_H_24_N_3_F_3_O_3_.HCl) C, 58.24; H, 5.04; N, 8.48. Found: C, 57.87; H, 4.92; N, 8.21. 

## 4. Conclusions 

The biological evaluation showed a broad range of activities, thereby offering new structural hits for future chemical pharmacomodulation of 1-aryl-3-substituted propanol derivatives as a new therapeutic option for the treatment of malaria.

## Figures and Tables

**Table 1:**
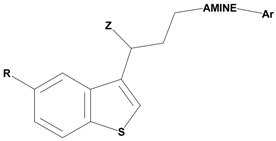
*In vitro* antimalarial activity of Benzo[b]thiophenyl derivatives versus Chloroquine Sensitive ·3D7 Strain of *Plasmodium falciparum*.

Compd.	R	Z	Amine	Ar	*P. falciparum* 3D7 IC_50_ μM
**1^a^**	F	C=O	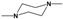	1-naphthyl	IN
**2 ^c^**	F	C=O	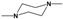	2-methoxyphenyl	IN
**3 ^b^**	H	C=O	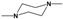	2-methoxyphenyl	IN
**4^a^**	F	C=O	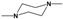	8-quinolyl	IN
**5^a^**	H	C=O	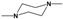	8-quinaldinyl	IN
**6**	H	C=O	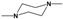	4-nitro-2-trifluromethyl phenyl	IN
**7**	H	C=O	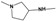	4-nitro-2-trifluromethyl phenyl	IN
**8**	H	C=O	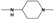	4-nitrophenyl	IN
**9**	H	C=O	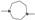	4-nitrophenyl	IN
**10**	H	C=O	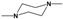	2-quinoxaline	IN
**11^a^**	H	OH	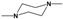	1-naphthyl	6.13
**12^a^**	F	OH	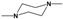	1-naphthyl	0.16
**13^b^**	H	OH	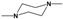	2-methoxyphenyl	1.07
**14^b^**	H	OH	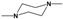	2-hydroxyphenyl	13.73
**15^c^**	F	OH	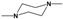	2-methoxyphenyl	0.33
**16**	F	OH	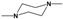	3-methoxyphenyl	1.67
**17^a^**	H	OH	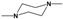	4-indolyl	6.99
**18^a^**	F	OH	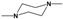	4-indolyl	3.81
**19^b^**	H	OH	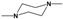	4-chlorophenyl	5.65
**20^a^**	H	OH	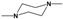	8-quinolyl	2.15
**21^a^**	F	OH	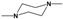	8-quinolyl	0.79
**22^a^**	H	OH	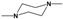	8-quinaldinyl	0,66
**23^a^**	F	OH	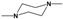	8-quinaldinyl	0.38
**24^a^**	H	OH	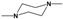	5-quinolyl	1.30
**25^a^**	H	OH	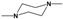	2-quinolyl	2.22
**26^a^**	H	OH	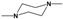	2,3-dihydro-1,4-benzodioxin-5-yl	3.56
**27^a^**	F	OH	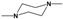	2,3-dihydro-1,4-benzodioxin-5-yl	0.90
**28**	H	OH	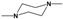	4-nitro-2-trifluromethyl phenyl	0.68
**29**	H	OH	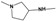	4-nitro-2-trifluromethyl phenyl	2.99
**30**	H	OH	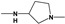	4-nitro-2-trifluromethyl phenyl	0.97
**31**	H	OH	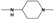	4-nitro-2-trifluromethyl phenyl	0.19
**32**	F	OH	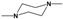	4-nitrophenyl	IN
**33**	H	OH	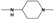	4-nitrophenyl	1.00
**34**	H	OH	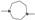	4-nitrophenyl	3.43
**35**	H	OH	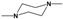	2-quinoxalinyl	0.62
Chloroq.					0.08

^a^ Drugs from [11]; ^b^ Drugs from [9]; ^c^ Drugs from [10]. IC_50_ is the 50% inhibitory concentration of the *in vitro* parasite growth. Each value is the mean of two experiments in triplicate. All the compounds with IC_50_ value higher than 20 µM was considered inactive (IN).

**Table 2:**
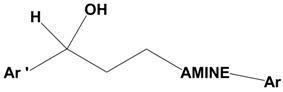
*In vitro* antimalarial activity of 1-aryl-3-substituted propanol derivatives versus Chloroquine Sensitive ·3D7 Strain of *Plasmodium falciparum.*

Compd.	Ar’	Amine	Ar	*P. falciparum* 3D7 IC_50_ μM
**36^b^**	3-thiophenyl	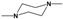	4-chlorophenyl	IN
**37^b^**	phenyl	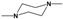	4-chlorophenyl	13.04
**38**	biphenyl	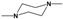	4-chlorophenyl	IN
**39^b^**	phenyl	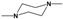	2-methoxyphenyl	IN
**40**	biphenyl	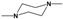	2-methoxyphenyl	14.63
**41**	3-indolyl	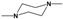	2-methoxyphenyl	IN
**42^b^**	2-naphthyl	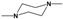	2-methoxyphenyl	0.83
**43^a^**	2,4-dimethylphenyl	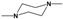	2-methoxyphenyl	16.91
**44**	6-methylnapht-2-yl	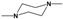	2-methoxyphenyl	9.64
**45**	2-naphthyl	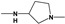	4-nitro-2-trifluromethyl phenyl	9.10
**46^a^**	2,4-dimethylphenyl	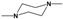	2-hydroxyphenyl	18.16
Chloroq.				0.08

^a^ Drugs from [11]; ^b^ Drugs from [9]. IC_50_ is the 50% inhibitory concentration of the *in vitro* parasite growth. Each value is the mean of two experiments in triplicate. All the compounds with IC_50_ value higher than 20 µM was considered inactive (IN).

**Table 3:**
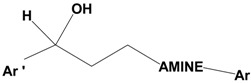
*In vivo* antimalarial activity of selected 1-aryl-3-arylamino propanol derivatives against *Plasmodium berghei* ANKA strain.

Compd.	Ar’	Amine	Ar	GIP of *P. bergei* at 50 mg/kg/day
**12**	5-F-Benzo[b]thiophenyl	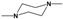	1-naphthyl	65,79
**23**	5-F-Benzo[b]thiophenyl	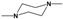	8-quinaldinyl	37,69
**15**	5-F-Benzo[b]thiophenyl	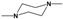	2-methoxyphenyl	24,73
**31**	Benzo[b]thiophenyl	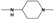	4-NO_2_-2-CF_3_-phenyl	Toxic
Chloroq.				100% at 10 mg/kg

GIP = Growth inhibition percentage of the rodent malaria parasite after a four-day treatment. 10 mg/kg/day of chloroquine, positive control, inhibited 100% the growth parasite. ^a^ Toxic = death of more than half of the animals in the tested group.
